# Steroidomics via Gas Chromatography–Mass Spectrometry (GC-MS): A Comprehensive Analytical Approach for the Detection of Inborn Errors of Metabolism

**DOI:** 10.3390/life15060829

**Published:** 2025-05-22

**Authors:** Francesco Chiara, Sarah Allegra, Simona Liuzzi, Maria Paola Puccinelli, Giulio Mengozzi, Silvia De Francia

**Affiliations:** 1 Department of Physics, University of Trento, 38123 Povo, Italy; francesco.chiara@unito.it; 2 Department of Clinical and Biological Sciences, University of Turin, 10043 Orbassano, Italy; silvia.defrancia@unito.it; 3 Clinical Biochemistry Laboratory “Baldi e Riberi”, A.O.U. Città della Salute e della Scienza di Torino, 10134 Turin, Italy; simonaliuzzi@hotmail.it (S.L.); mpuccinelli@cittadellasalute.to.it (M.P.P.); giulio.mengozzi@unito.it (G.M.)

**Keywords:** GC-MS, steroidomics, urinary steroids, inherited metabolic disorders

## Abstract

**Background:** Urinary steroid profiling plays a key role in the diagnosis of inherited and acquired endocrine disorders. Despite the proven diagnostic value of gas chromatography–mass spectrometry (GC-MS), standardized and clinically validated protocols for extended steroid panels remain limited. **Methods:** We developed and validated a GC-MS method for the quantification of 32 urinary steroid metabolites, including androgens, estrogens, progestins, glucocorticoids, and mineralocorticoids. Sample preparation involved solid-phase extraction, enzymatic hydrolysis, and dual derivatization, followed by chromatographic separation and mass detection under full scan mode. Validation followed ICH M10 guidelines. **Results:** The method demonstrated high selectivity, accuracy (within ±15%), and precision (CV% < 15%) across three QC levels. Limits of Quantification were estimated using the Hubaux–Vos approach and were suitable for detecting both physiological and pathological steroid concentrations. Robustness and matrix effect tests confirmed the method’s reliability and reproducibility. **Conclusions:** This GC-MS protocol enables comprehensive urinary steroid profiling and calculation of diagnostic ratios for inborn errors of steroid metabolism and endocrine disorders. The method is suitable for clinical application and future integration into personalized medicine workflows.

## 1. Introduction

The analysis of steroid metabolites in biological fluids is crucial for understanding endocrine function and diagnosing metabolic disorders. Due to the structural complexity and wide range of concentrations of these compounds, sophisticated analytical techniques are required. While early approaches relied on less specific methods, the advent and coupling of gas chromatography with electron ionization mass spectrometry (GC-EI-MS) in the mid-20th century [1] revolutionized the field, enabling the detailed analysis and “profiling” of complex steroid mixtures in urine following enzymatic hydrolysis and derivatization [2]. This technique rapidly became a cornerstone for steroid metabolomics [3,4], particularly valued for its high chromatographic resolution, essential for separating numerous steroid isomers, and for providing highly characteristic EI mass spectra suitable for confident identification via comparison with extensive mass spectral libraries. Although liquid chromatography–mass spectrometry (LC-MS) techniques, including electrospray ionization (ESI), also developed and became prominent, offering distinct advantages particularly for polar and conjugated metabolites and high-throughput analysis, the application to comprehensive steroid panels evolved over time [5,6]. Nevertheless, GC-MS after appropriate sample preparation remains a powerful and often preferred method for comprehensive profiling of the less polar, unconjugated urinary steroid metabolites where maximizing chromatographic separation and obtaining definitive spectral information for identification are paramount for resolving complex diagnostic profiles relevant to inborn errors of metabolism and other endocrine disorders [4,6,7,8,9].

In parallel with technological advancements, the clinical utility of urinary steroid profiling has expanded significantly. Modern mass spectrometry-based platforms now allow for the simultaneous quantification of dozens of steroid metabolites, enabling not only the diagnosis of enzymatic defects but also the evaluation of functional activity along steroidogenic pathways [3,10]. In contrast to isolated single-analyte testing, the profiling approach captures complex biochemical signatures, making it a valuable tool for both diagnostic and prognostic purposes.

More recently, urinary steroid metabolomics has emerged as a powerful tool not only in rare metabolic syndromes but also in common endocrine conditions, including disorders of the female reproductive system. Alterations in the androgen, estrogen, and corticosteroid pathways are observed in a range of conditions, from polycystic ovary syndrome and endometriosis to adrenal tumors [11,12]. These alterations often manifest as characteristic shifts in the relative abundance of specific urinary metabolites, such as etiocholanolone, androsterone, tetrahydro-cortisol derivatives, and their hydroxylated or keto counterparts.

Despite the growing importance of these profiles in clinical endocrinology, standardized, validated methods for the simultaneous quantification of extended urinary steroid panels remain limited. GC-MS remains a cornerstone and often considered a reference method for comprehensive steroid profiling, particularly for isomeric compounds and low-abundance metabolites [4,6,7,8]. However, most available protocols are either insufficiently validated for clinical translation or restricted to limited subsets of steroids.

In this context, we report the analytical validation of a GC-MS method designed to quantify a comprehensive panel of 32 urinary steroid metabolites, including key androgens, estrogens, pregnanes, and corticosteroids. This method was developed to address current gaps in routine diagnostics, integrating both historical markers of congenital metabolic disorders and emerging indicators of reproductive and adrenal dysfunction. The final profile, detailed in Table 1, Table 2, Table 3, Table 4 and Table 5 was selected to maximize clinical relevance based on both the legacy literature and recent metabolomic evidence [13].

## 2. Materials and Methods

### 2.1. Chemicals

Unless otherwise specified, all reagents were purchased from Merck (Darmstad, Germany). In particular n-hexane, ethyl acetate, methanol, isopropanol, and anhydrous pyridine were GC-MS grade; silylating mixture II according to Horning [14] was constituted from *N*,*O*-Bis(trimethylsilyl)acetamide, chlorotrimethylsilane, and 1-(trimethylsilyl)imidazole mixture, with volumetric ratio as BSA+TMCS+TMSI 3:2:3. The Sigmatrix Urine Diluent (SUD), non-biological diluent that mimics human urine, was used to prepare simulated sample. The 18 MΩ water was produced with Thermo Scientific™ (Waltham, MA, USA) Barnstead™ GenPure™ Pro; the standard powders of each steroid were purchased from Steraloids Inc., based in Newport, (RI, USA). The Strata C18-E SPE cartridges were obtained from Phenomenex (Torrance, CA, USA). Beta-glucuronidase/sulfatase enzyme from Helix pomatia (Type H-2, aqueous solution, Product Number G0876) was purchased from Sigma-Aldrich (St. Louis, MO, USA). According to the Certificate of Analysis, the lot used had a glucuronidase activity of 85,707 units/mL and a sulfatase activity of 778 units/mL.

### 2.2. Stock Solutions, Standards (STDs), and Quality Controls (QCs)

- Individual stock solutions: Each steroid standard was dissolved according to the manufacturer’s certificate of analysis to prepare a stock solution at the recommended concentration.
- Working solutions: Stock solutions were diluted in isopropanol to obtain individual working solutions for each analyte.
- Mixed stock solution: Equal volumes of each individual working solution were combined to prepare a mixed stock solution containing all target analytes.
- Calibration solutions: The mixed stock solution was serially diluted in isopropanol to generate six calibration levels.
- Spiked samples: Each calibration level was diluted 1:10 in surrogate urine diluent (SUD) to prepare matrix-based spiked samples for calibration.
- Quality control (QC) samples: Three QC levels (low, medium, high) were prepared in SUD in accordance with ICH M10, Section 3.2.5.1.
- Blank sample: Prepared by mixing deionized water and SUD in a 1:10 volume ratio without any added analytes.
- Buffer A (3 M Acetate Buffer, pH 4.6)—A solution (A1) of 3 M acetic acid is prepared by diluting 43.27 mL of glacial acetic acid (d = 1.04) to a final volume of 250 mL with deionized water. A solution (B1) of 3 M sodium acetate is prepared by dissolving 61.5 g of anhydrous salt or 102 g of trihydrate salt in 250 mL of deionized water. Mix 152 mL of A1 with 147 mL of B1. The pH is then measured.
- Buffer B (0.2 M Acetate, pH 4.6): A solution (A2) of 0.2 M acetic acid is prepared by diluting 5.75 mL of glacial acetic acid (d = 1.04) to a final volume of 500 mL with deionized water. A solution (B2) of 0.2M sodium acetate is prepared by dissolving 8.2 g of anhydrous salt or 13.6 g of trihydrate salt in 500 mL of deionized water. Mix 255 mL of A2 with 245 mL of B2. The pH is then checked.
- Bicarbonate Buffer (pH 10.5): A solution (A) of 0.1 M sodium carbonate is prepared by dissolving 10.6 g of anhydrous Na_2_CO_3_ in 1 L of deionized water. A solution (B) of 0.1M sodium bicarbonate is prepared by dissolving 4.200 g of anhydrous salt (MW = 84.007) in 500 mL of deionized water. Mix 771.5 mL of solution (A) with 228.5 mL of solution (B) and adjust the final volume to 1L with deionized water. The pH is checked and, if necessary, adjusted by adding a few drops of 2N sodium hydroxyde.
- Internal Standard (IS) Solution: A stock solution of stigmasterol was prepared by dissolving 18 mg of standard powder in 10 mL of isopropanol. The working solution was then prepared by diluting the stock solution 1:100 in methanol.
- Methoxyamine (MOX) solution: 100 mg of methoxyamine hydrochloride was dissolved in 10 mL of anhydrous pyridine. The prepared solution is stored in an amber tube, protected from light, at −20 °C.
- 0.05 M Sulfuric acid solution: It was prepared by diluting 250 μL of fuming H_2_SO_4_ in 93 mL of deionized water.
- Acidified water: It was prepared by diluting 3 mL of glacial acetic acid in 1 L of deionized water.

### 2.3. Preparation of Urinary Samples for GC-MS Analysis of the Steroids Profile

#### 2.3.1. *Total Steroids Extraction*

Before proceeding with the extraction, the samples must be vortexed and centrifuged. If they are frozen (stored at −80 °C), they should be properly thawed. Then, a series of new SPE columns, numbered according to the analytical sequence, should be set up on the SPE manifold and conditioned as follows: 3 mL of methanol is dispensed, followed by 3 mL of acidified water; the vacuum is applied, and the eluate is discarded. The flow is stopped by closing the valves when the meniscus is about 3–5 mm from the upper portion of the stationary phase.

At this point, using a series of plastic tubes with caps, numbered in the same way as the SPE columns, 5 mL of urine is dispensed into each tube. Then, 2 mL of buffer A is added, vortexed, and centrifuged. Next, 100 μL of internal standard (IS) solution (stigmasterol, stored at −20 °C) is dispensed into each column, and the buffered urine solution is transferred from the tubes to the columns. Percolation is carried out through the SPE columns by applying a vacuum to maintain a flow rate not exceeding 3 mL/min, and the eluate is discarded.

At this point, a series of glass tubes, numbered like the SPE columns, is placed under the outlet nozzles. Elution is performed twice using 2 mL of methanol each time, percolating at a moderate flow rate and collecting the eluate in the previously prepared tubes. The eluate is then evaporated under a nitrogen stream in a water bath at 45 °C until the volume is reduced to approximately 500 μL. This partial evaporation, avoiding complete dryness, to avoid potential degradation or loss of thermolabile derivatized metabolites and facilitates reconstitution in the subsequent enzymatic hydrolysis buffer. The extraction procedure can be interrupted at this stage by freezing the eluate and storing the SPE columns in the refrigerator for up to 24 h. The volume of buffer used in the subsequent step is sufficient to dilute the residual methanol, ensuring optimal activity of the enzyme mixture.

#### 2.3.2. *Enzymatic Hydrolysis of Conjugates and Extraction*

To the concentrated eluate obtained, 5 mL of buffer B and 200 μL of glucuronidase/ sulfatase are added. The tubes are then incubated for 3 h at 55 °C in a thermostated water bath. After incubation, the samples are vortexed and centrifuged.

At this point, the previously used columns are reconditioned by dispensing 3 mL of methanol followed by 3 mL of acidified water, ensuring that the eluate is percolated and discarded by applying the necessary vacuum. The solvent flow is stopped under the same conditions described in the previous section.

The supernatant from each tube is then transferred to the corresponding numbered column and percolated under vacuum, ensuring that the elution flow rate does not exceed 3 mL/min. The eluate is discarded, and the SPE columns are eluted twice with 2 mL of ethyl acetate, maintaining a moderate elution flow rate and collecting the eluate in the prenumbered tubes.

At this stage, 2 mL of bicarbonate buffer (pH 10.5) is dispensed into each tube, capped, and vortexed for 30 s, then centrifuged. In a third set of correspondingly numbered tubes, the supernatant (organic phase) is transferred and evaporated to dryness under a nitrogen stream in a water bath at 45 °C.

If necessary, this second preparative phase can be interrupted, resuming the process by reconstituting the dried residue with 50 μL of methanol and storing it at −20 °C for up to 24 h.

#### 2.3.3. *Double Derivatization of Free Steroids*

**Figure 1:**
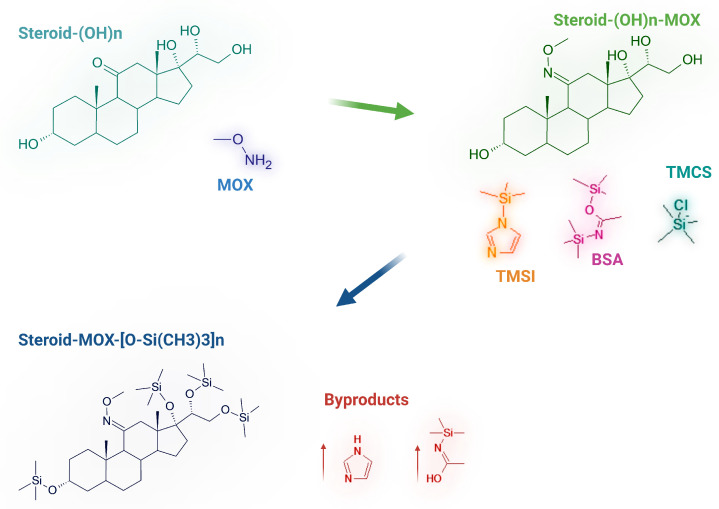
Reaction scheme of the derivatization process for hydroxysteroids: methoximation (MOX) followed by silylation using a mixture of BSA (*N*,*O*-Bis(trimethylsilyl)acetamide), TMCS (chlorotrimethylsilane), and TMSI (trimethylsilylimidazole). The resulting derivatives are trimethylsilyl (TMS)-protected steroids, as shown in the final structure.

To each tube, 100 μL of MOX solution in pyridine is added. At this stage, it is important to cap the tubes with clean and dry caps; vortex, invert, and incubate for 1 h at 80 °C. Then, 100 μL of derivatizing reagent F is added to each tube; vortex, invert, and incubate for 4 h at 100 °C. This phase can be interrupted by freezing the tubes at −20 °C, knowing that the derivatized compound is stable under these conditions for 15 days. The overall derivatization mechanism, including both methoximation and silylation steps with the BSA+TMCS+TMSI mixture, is illustrated in Figure 1.

Next, 3 mL of n-hexane and 2 mL of 0.05 M sulfuric acid are added. Vortex for 30 s until the solution is clear, then proceed to remove the aqueous phase at the bottom of the tube using a Pasteur pipette. Add 2 mL of 0.05 M sulfuric acid again, vortex the tubes for 30 s, and then centrifuge.

Afterward, transfer the upper organic phase obtained into a new glass tube. The solvent is then evaporated to dryness in a water bath at 45 °C under nitrogen. The residue is reconstituted with 120 μL of n-hexane, vortexed well, and 50 μL is transferred into a vial for GC-MS injection.

### 2.4. *GC-MS Analysis*

The analyses were performed using a Hewlett Packard HP 6890 gas chromatograph (Agilent Technologies Inc., Santa Clara, CA, USA) coupled with an HP 5973 mass selective detector (MSD) (Agilent Technologies Inc., Santa Clara, CA, USA). Chromatographic separation was achieved using a CPS Analitica CC-5 MS capillary column (CPS Analitica, Milano, Italy) (50 m length, 0.25 mm internal diameter, 0.25 μm film thickness, crossbond), with helium as the carrier gas under constant flow conditions (initial flow: 0.6 mL/min; inlet pressure: 7.65 psi). The maximum column temperature was 350 °C.

The GC oven temperature program was as follows: initial temperature 50 °C (held for 0 min), then ramped at 50 °C/min to 230 °C, 0.4 °C/min to 250 °C (held for 5 min), 20 °C/min to 270 °C, and 50 °C/min to 285 °C (held for 30 min). Total run time was 89.9 min.

This temperature program, including the multiple ramps, was empirically optimized to achieve maximal chromatographic resolution for the complex panel of steroid metabolites, ensuring adequate separation of critical isomers across the entire elution range.

The injector was operated in splitless mode at 285 °C. The injection volume was 2.0 μL, using a 10 μL syringe. The purge flow was set to 50.5 mL/min, with a purge activation delay of 2.5 min. A gas saver flow of 15 mL/min was used after 2 min.

The MS detector operated in scan mode with a solvent delay of 20 min, scanning from *m*/*z* 75 to 700. The ion source and quadrupole temperatures were set at 230 °C and 150 °C, respectively. Electron ionization was used, and mass calibration was verified before acquisition.

Chromatographic data were automatically integrated. Compounds were identified by comparing their retention times and acquired full mass spectra with those contained in the NIST 05 Mass Spectral Library and a dedicated in-house library (STEROIDI.L) generated using authentic standards of all target analytes under the method’s specific conditions. Quantification was performed by integrating the area of specific ions (target ion (Tgt), and qualifying ions (Q1, Q2, Q3)) extracted from the full scan data for each analyte. Calibration curves were generated using multiple concentration levels (6 points) covering the relevant range, and quality control samples were analyzed at three levels of concentration. Blank samples were also included in each analytical batch. Quantification was performed using linear regression models, applying quality acceptance criteria in accordance with international validation guidelines (ICH M10).

### 2.5. Method Performance: Selectivity, Specificity, Accuracy, Precision, and Sensitivity Parameters

Selectivity was evaluated using a blank surrogate urine matrix (Sigmatrix Urine Diluent, SUD) processed in the same way as study samples. The absence of interference was confirmed by analyzing at least six blank samples, ensuring that any signal at the retention time of the analytes and internal standards was <20% of the LLOQ response for each analyte and <5% for the internal standard, in accordance with ICH M10 guidelines.

Specificity was assessed by verifying the absence of significant interference from structurally related compounds, endogenous substances, or degradation products. No significant back-conversion or cross-talk between analytes was observed under the analytical conditions applied.

Accuracy and precision were determined by analyzing five replicates of QC samples at three concentration levels (low, medium, and high) across the calibration range, in multiple analytical runs. QCs were prepared by diluting reference mix solutions (10× in solvent) in a 1:10 volume ratio using SUD. Acceptance criteria followed ICH M10: accuracy within ±15% of nominal values (±20% for LLOQ), and precision (%CV) not exceeding 15% (20% for LLOQ), both within- and between-run.

Limits of Detection (LOD) and Limits of Quantification (LLOQ) were estimated according to the statistical model proposed by Hubaux and Vos [15], based on the confidence intervals of the regression line derived from six calibration levels. This method, based on the confidence intervals of the regression line derived from six calibration levels, allows for defining two kinds of lower limits from a statistical point of view: a decision limit (yc = LLOQ), representing the lowest signal that can be statistically distinguished from the background, and a detection limit (yD = LOD), corresponding to the content under which, a priori, any sample may erroneously be taken for a blank. These limits were calculated considering predefined type I and type II error probabilities, and the data are reported in Table 6, Table 7, Table 8, Table 9, Table 10, Table 11, Table 12, Table 13, Table 14 and Table 15.

### 2.6. Analyte Recovery and Extraction Efficiency Evaluation

Recovery (R) was assessed by comparing the peak areas of analytes spiked into SUD before extraction with those spiked after extraction (post-extraction spike), at low and high QC levels.

Extraction efficiency (EE) was evaluated by comparing the response of extracted samples to that of equivalent neat solutions prepared at the same concentrations. All measurements were performed in triplicate, and acceptable values for R and EE were within 85–115% of the nominal response.

### 2.7. Evaluation of Method Robustness and Matrix Interference

Robustness of the method was evaluated by intentionally introducing minor variations in analytical conditions, including injector temperature, carrier gas flow rate, and sample preparation timing. Under all tested conditions, the method maintained acceptable levels of accuracy and precision, confirming its robustness. Although the current validation was conducted using a surrogate matrix, the method is considered suitable for future clinical application, provided that equivalent performance is confirmed in real urine samples.

Matrix effects were assessed using Sigmatrix Urine Diluent, a synthetic surrogate matrix with a controlled and reproducible composition. Given its high lot-to-lot consistency, matrix effect evaluation was performed using multiple replicates of low and high QC samples from a single validated lot. The internal standard-normalized matrix factor (MF) showed a %CV below 15%, indicating minimal ion suppression or enhancement and confirming the suitability of the matrix for method validation purposes.

### 2.8. Stability of Analytes and Samples

Stability of the analytes was evaluated under different conditions:

- Freeze-thaw stability was tested over three cycles using low and high QCs stored at −20 °C between cycles.
- Short-term (bench-top) stability was assessed by keeping spiked QCs at room temperature for the duration of sample preparation and analysis.
- Long-term stability was evaluated for QCs stored at −20 °C for a period exceeding the expected study sample storage time.
- Post-preparative (autosampler) stability was tested by re-injecting extracted samples after storage at the autosampler temperature for the full runtime.

In all cases, the mean measured concentrations remained within ±15% of the nominal value, confirming stability. Stock and working solutions were also tested and shown to be stable under the applied storage conditions.

## 3. Results

### 3.1. Specificity, Selectivity, Accuracy, Precision, and Limits of Quantification and Detection

The total ion chromatogram (TIC) and individual mass spectra acquired for each analyte confirmed high specificity of the method, with full chromatographic separation and no evidence of cross-talk or overlapping peaks. Mass spectra showed the expected fragmentation patterns for all target compounds and were consistent with reference spectra from both commercial and in-house libraries.

Selectivity was verified by the absence of interfering peaks in the blank surrogate matrix (Sigmatrix Urine Diluent) at the retention times of the analytes and internal standard (Stigmasterol). No matrix components exceeded 20% of the analyte response at the LLOQ level.

Accuracy and precision were confirmed across the entire analytical range. All analytes met the acceptance criteria established by ICH M10, with intra-assay and inter-assay coefficients of variation (CV%) below 15% at each QC level (and below 20% at LLOQ). The method demonstrated acceptable performance for all validated analytes in terms of repeatability and reproducibility, as defined by CV% values across three quality control (QC) levels (low, medium, high).

Repeatability (Rep.) and reproducibility (Repr.) data for each steroid class are presented separately in Table 16, Table 17, Table 18 and Table 19. Specifically, Table 16 summarizes the performance of progestins, Table 17 for mineralocorticoids, Table 18 for glucocorticoids, Table 20 for androgens, and Table 19 for estrogens. In each table, Rep. and Repr. are expressed as CV% and refer to intra-assay and inter-assay precision, respectively.

All values obtained were below the acceptance criteria of 15% CV for medium and high QC levels, and 20% for the lower limit of quantification (LLOQ), in accordance with the ICH M10 guidelines. These results confirm the robustness and reproducibility of the method for each metabolite tested.

The internal standard (IS), stigmasterol, was monitored to ensure consistency in sample preparation and instrument performance but was excluded from the QC-based CV calculations. The precision and retention time data demonstrate the reliability and robustness of the method for clinical application.

The Limits of Detection (LOD) and Quantification (LLOQ) were calculated according to the statistical approach proposed by Hubaux and Vos. All analytes displayed sufficient signal-to-noise ratios to meet detection criteria, with LLOQs compatible with expected urinary concentrations under physiological and pathological conditions.

**Figure 2:**
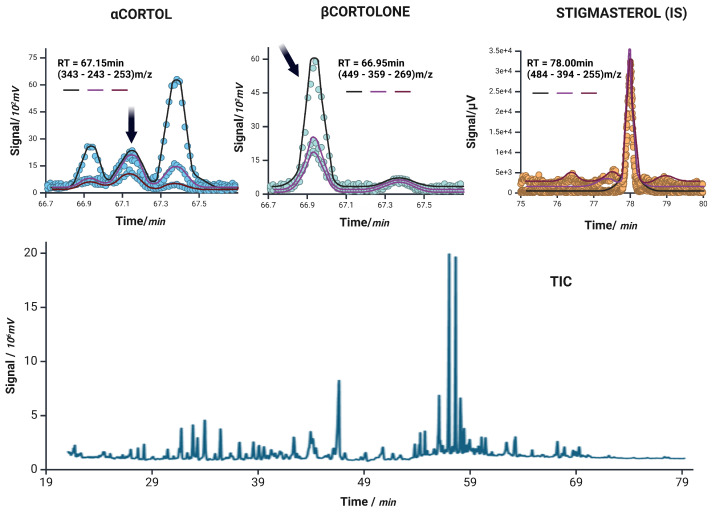
Extracted ion chromatograms for α-Cortol, β-Cortolone, and the internal standard stigmasterol (IS) are shown in the top row. Each panel displays the ion currents of the monitored transitions: target ion (black), qualifier 1 (magenta), and qualifier 2 (purple), corresponding to *m*/*z* values used in the acquisition. Dots represent the raw signal intensity, while the overlaid solid lines reflect the smoothed chromatographic profiles of each individual ion current. The bottom panel shows the unprocessed total ion current (TIC) chromatogram of a representative sample, illustrating the overall complexity and retention time distribution of the steroidal metabolites analyzed.

According to ICH M10 guidelines, selectivity is confirmed when the response of blank samples at the analyte retention time is less than 20% of the response at the lower limit of quantification (LLOQ), and less than 5% for internal standards. In our method, at least six blank samples were analyzed and no significant signals were observed. The representative extracted ion chromatograms (EICs) shown in Figure 2 demonstrate the high selectivity and lack of interference in the detection of target analytes.

### 3.2. Recovery and Extraction Efficiency

Although absolute recovery was not the primary endpoint of this method validation, the consistency of analytical response across QC levels and minimal variability in replicate samples support an efficient extraction protocol. Peak areas of spiked samples showed minimal deviation from expected values, confirming stable analyte behavior through sample handling and derivatization.

### 3.3. Robustness and Matrix Effect Evaluation

**Figure 3:**
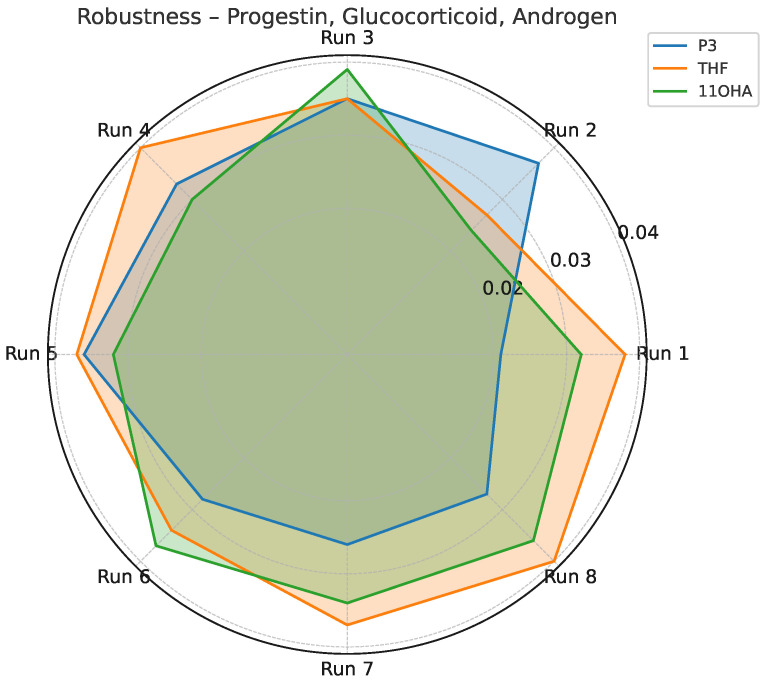
Standard deviation profile for P3, THF, and 11OHA across robustness testing conditions (Yates matrix).

**Figure 4:**
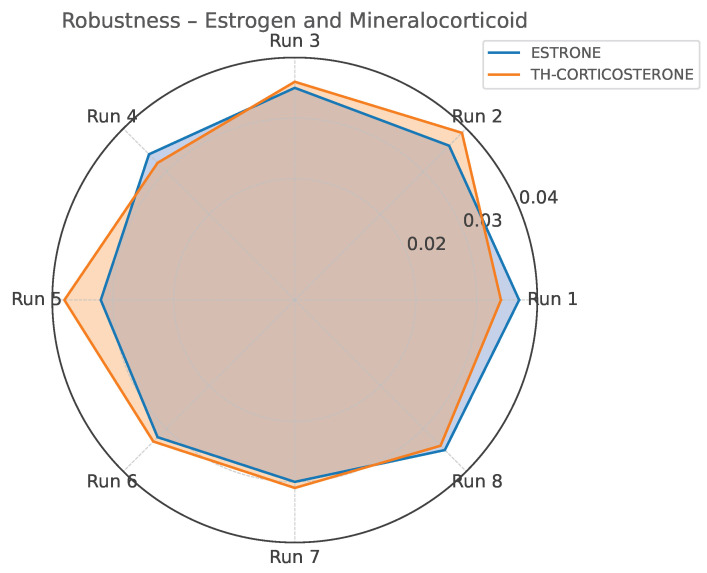
Standard deviation profile for Estrone and TH-Corticosterone across robustness testing conditions (Yates matrix).

Robustness was tested using a factorial design (Yates matrix) by varying key instrumental parameters, including injector temperature, carrier gas flow rate, and splitless time. The F-test applied to compare the standard deviations of replicate measurements under normal and stressed conditions revealed no statistically significant differences (F < F_crit_ = 2.87) for any analyte. The only borderline case was observed for 11ß-OH-androsterone, which nonetheless remained within acceptable precision limits, confirming overall method robustness. Representative results are illustrated in Figure 3 and Figure 4.

Matrix effects were assessed in the surrogate matrix used for method development. Due to the standardized and defined composition of Sigmatrix Urine Diluent, consistent signal responses were observed across replicate preparations. IS-normalized matrix factors showed a coefficient of variation below 15%, fulfilling the requirements for bioanalytical application.

### 3.4. Stability

Stability was evaluated under multiple storage and processing conditions. All analytes demonstrated stability in the autosampler (post-preparative stability), after three freeze–thaw cycles, and during short-term benchtop exposure. No significant degradation or loss of signal was observed. Analyte responses remained within ±15% of nominal values across all tested conditions, confirming suitability for routine use in a clinical or research setting.

## 4. Discussion

The method here demonstrates excellent analytical performance in terms of sensitivity, reproducibility, and robustness, aligning with the acceptance criteria of ICH M10. GC-MS has long been recognized as the gold standard for urinary steroid profiling, enabling the detection of complex metabolic signatures in endocrine and metabolic diseases [4].

Our assay quantifies 32 urinary steroids across five major hormonal classes—progestins, mineralocorticoids, glucocorticoids, androgens, and estrogens—with precision values consistently below 15% CV (Table 16, Table 17, Table 18 and Table 19). The performance was maintained even under conditions of analytical stress, as shown by robustness testing (Figure 3 and Figure 4). These findings support the suitability of the method for routine application in clinical laboratories.

Importantly, the method also allows for the calculation of diagnostic ratios between steroid metabolites. These ratios—first proposed by Shackleton and later refined by Rousson et al. and Ackermann et al.—serve as functional biomarkers reflecting enzymatic activities within steroidogenic pathways [2,9,16]. Examples include the ET/AN ratio (5β/5α), 11β-hydroxylase deficiency indices (e.g., THS/THF), or 21-hydroxylase markers (17HP/PTONE), which are crucial in the differential diagnosis of congenital adrenal hyperplasia (CAH) and other inborn errors of metabolism [17].

The method’s application is not limited to rare diseases. Recent data support its utility in subtyping Cushing’s syndrome [18], as well as in steroid-related nephrotic syndrome and reproductive endocrinology [6]. The inclusion of estrogens and progestins, often neglected in routine workflows, expands its potential utility in assessing disorders such as PCOS, endometriosis, or hormonal therapies.

Finally, the minimal matrix effect and use of a stable surrogate matrix (SUD) confirm the method’s compatibility with high-throughput sample processing and quantitative metabolomics workflows. Its design is well suited to future integration with multi-omics platforms and longitudinal patient stratification approaches [7,19].

## 5. Conclusions

We developed and validated a GC-MS method for the quantitative profiling of urinary steroid metabolites that meets international analytical standards. The method allows for the simultaneous measurement of 36 key steroids, with high precision and robustness, and enables the calculation of clinically relevant diagnostic ratios.

By integrating classic metabolic profiling with updated diagnostic criteria, this method paves the way for broader implementation of urinary steroid metabolomics in clinical endocrinology. Its application to real clinical samples will further validate its use in the differential diagnosis of endocrine and metabolic disorders, particularly in contexts where steroidogenic enzyme defects are suspected. The results presented support its suitability for routine application and for future integration into personalized medicine workflows.

## Figures and Tables

**Table 1:** Steroid metabolites in the progestins group with retention times and associated clinical conditions.

Analyte	Abbreviation	RT (min)	Associated Conditions
17OH-Pregnanolone	17HP	45.72	Elevated in CAH due to 21-hydroxylase or 11β-hydroxylase deficiency.
Pregnanediol	P2	50.66	Major metabolite of progesterone; increased in 21-hydroxylase deficiency (classic CAH).
Pregnentriol	5PT	60.29	Marker of 3β-hydroxysteroid dehydrogenase (3β-HSD) deficiency; increased in non-classic CAH variants.
Pregnantriolone	PTONE	60.67	Derived from 17-hydroxyprogesterone (17OHP); elevated in 21-hydroxylase deficiency. Often reported with pregnanetriol.
Pregnantriol	PT	61.92	Major urinary metabolite of 17OHP; markedly elevated in 21- and 11β-hydroxylase deficiency (CAH).

**Table 2:** Steroid metabolites in the mineralocorticoids group with retention times and associated clinical conditions.

Analyte	Abbreviation	RT (min)	Associated Conditions
TH-11-Deoxycorticosterone	THDOC	60.38	Metabolite of DOC; elevated in 11β-hydroxylase deficiency, sometimes in AME.
5α-TH-Corticosterone	5αTHB	64.04	Metabolite in the aldosterone biosynthesis pathway.
TH-Corticosterone	THB	64.80	Aldosterone pathway metabolite.
TH-11-Dehydrocorticosterone	THA	65.04	Derived from 11-dehydrocorticosterone; altered in 11β-hydroxylase deficiency.

**Table 3:** Steroid metabolites in the glucocorticoids group with retention times and associated clinical conditions.

Analyte	Abbreviation	RT (min)	Associated Conditions
11-Keto-Etiocholanolone	11ketoET	42.88	Metabolite of cortisone pathway.
11β-OH-Etiocholanolone	11βOHET	45.79	Elevated in 11β-hydroxylase deficiency.
TH-11-Deoxycortisol	THS	57.39	Highly elevated in 11β-hydroxylase deficiency.
TH-Cortisone	THE	63.03	Cortisone metabolite.
TH-Cortisol	THF	63.80	Cortisol metabolite.
5α-TH-Cortisol	5αTHF	64.33	Cortisol metabolite; used in 5α/5β ratio for ACRD.
ββ-Cortol	βCortol	65.10	Cortisol metabolite; altered in ACRD.
α-Cortolone	αCortolone	65.40	Cortisol metabolite; altered in ACRD.
β-Cortolone	βCortolone	66.75	Cortisol metabolite; altered in ACRD.
α-Cortol	αCortol	66.95	Cortisol metabolite; altered in ACRD.
Cortisol	F	77.63	Primary glucocorticoid excreted in urine.
20α-Dihydro-Cortisol	20αDHF	81.19	Minor cortisol metabolite.

**Table 4:** Steroid metabolites in the androgens group with retention times and associated clinical conditions.

Analyte	Abbreviation	RT (min)	Associated Conditions
5α-Androstanediol	5αADIOL	30.50	Androgen metabolite.
Androsterone	AN	34.24	Elevated in CAH and androgen excess. ET/AN ratio is informative.
Δ5-Androstenediol	D5-ADIOL	34.37	Altered in 3β-HSD deficiency.
Etiocholanolone	ET	35.40	Elevated in CAH and androgen excess. ET/AN ratio is informative.
Dehydroepiandrosterone	DHEA	38.25	Precursor/metabolite in adrenal androgen pathway; elevated in CAH and adrenal tumors.
11β-OH-Androsterone	11βOHAN	40.00	Derived from cortisol metabolism; elevated in 11β-hydroxylase deficiency.
16α-OH-DHEA	16αOHDHEA	47.90	DHEA pathway metabolite.
Androstenetriol	D5-ATRIOL	53.02	Altered in 3β-HSD deficiency.

**Table 5:** Steroid metabolites in the estrogens group with retention times and associated clinical conditions.

Analyte	Abbreviation	RT (min)	Associated Conditions
Estrone	E1	43.39	Estrogen metabolite.
Estriol	E3	59.14	Estrogen metabolite; also a pregnancy marker.
17β-Estradiol	E2	76.06	Estrogen metabolite.

**Table 6:** Slope and intercept parameters with 95% confidence intervals for progestins (GC-MS).

Analytes	Slope (m)	CI_low_(m)	CI_high_(m)	Intercept (q)	CI_low_(q)	CI_high_(q)
P2	0.0326	0.0309	0.0343	0.0913	0.0529	0.1298
5PT	0.0111	0.0107	0.0115	0.1459	0.1368	0.1550
17HP	0.0337	0.0330	0.0345	0.1922	0.1754	0.2090
PT	0.0153	0.0150	0.0156	0.1379	0.1311	0.1447
PTONE	0.0686	0.0656	0.0717	0.0970	0.0282	0.1658

**Table 7:** LOD, LOQ, LLOQ, recovery, and $R^{2}$ for progestins (GC-MS).

Analytes	LOD (μg/mL)	LOQ (μg/mL)	LLOQ (μg/mL)	Recovery (%)	R^2^
P2	2.172	6.581	0.010	97.7100	0.9986
5PT	1.507	4.567	0.010	109.3800	0.9993
17HP	0.919	2.785	0.010	107.7000	0.9997
PT	0.818	2.478	0.010	102.5500	0.9998
PTONE	1.848	5.599	0.010	110.3000	0.9990

**Table 8:** Slope and intercept parameters with 95% confidence intervals for mineralocorticoids (GC-MS).

Analytes	Slope (m)	CI_low_(m)	CI_high_(m)	Intercept (q)	CI_low_(q)	CI_high_(q)
THDOC	0.0490	0.0477	0.0504	0.0068	−0.0234	0.0369
THA	0.0784	0.0764	0.0803	0.1371	0.0938	0.1804
5αTHB	0.0483	0.0469	0.0497	0.0611	0.0299	0.0923
THB	0.0568	0.0548	0.0589	0.0967	0.0502	0.1431

**Table 9:** LOD, LOQ, LLOQ, recovery, and $R^{2}$ for mineralocorticoids (GC-MS).

Analytes	LOD (μg/mL)	LOQ (μg/mL)	LLOQ (μg/mL)	Recovery (%)	R^2^
THDOC	1.132	3.431	0.010	99.2100	0.9996
THA	1.018	3.086	0.010	103.2100	0.9997
5αTHB	1.190	3.606	0.010	94.9100	0.9996
THB	1.505	4.561	0.010	112.0600	0.9993

**Table 10:** Slope and intercept parameters with 95% confidence intervals for glucocorticoids (GC-MS).

Analytes	Slope (m)	CI_low_(m)	CI_high_(m)	Intercept (q)	CI_low_(q)	CI_high_(q)
THS	0.0469	0.0463	0.0474	0.0590	0.0460	0.0721
F	0.0762	0.0734	0.0790	0.1532	0.0911	0.2152
THF	0.0099	0.0090	0.0108	0.0277	0.0084	0.0469
5α-THF	0.0321	0.0303	0.0339	0.1534	0.1135	0.1932
β-Cortol	0.0412	0.0396	0.0427	0.1401	0.1046	0.1757
α-Cortol	0.0276	0.0269	0.0284	0.1468	0.1297	0.1638
20α-DHF	0.0242	0.0207	0.0276	0.2051	0.1275	0.2827
11β-OHET	0.0396	0.0378	0.0415	0.1408	0.0997	0.1819
THE	0.0178	0.0167	0.0189	0.1304	0.1052	0.1556
α-Cortolone	0.0540	0.0530	0.0551	0.0312	0.0069	0.0554
β-Cortolone	0.0151	0.0143	0.0160	0.0769	0.0575	0.0962
11ketoET	0.0289	0.0279	0.0299	0.1169	0.0934	0.1404

**Table 11:** LOD, LOQ, LLOQ, recovery, and $R^{2}$ for glucocorticoids (GC-MS).

Analytes	LOD (μg/mL)	LOQ (μg/mL)	LLOQ (μg/mL)	Recovery (%)	R^2^
THS	0.513	1.554	0.010	96.6000	0.9999
F	1.500	4.545	0.010	90.9700	0.9993
THF	3.577	10.839	0.010	108.9800	0.9961
5α-THF	2.285	6.925	0.010	91.1600	0.9984
β-Cortol	1.591	4.822	0.010	98.6300	0.9992
α-Cortol	1.137	3.444	0.010	90.2400	0.9996
20α-DHF	5.919	17.936	0.010	114.8800	0.9895
11β-OHET	1.912	5.794	0.010	99.5000	0.9989
THE	2.617	7.930	0.010	94.2900	0.9979
α-Cortolone	0.826	2.503	0.010	90.6800	0.9998
β-Cortolone	2.352	7.126	0.010	111.3100	0.9983
11ketoET	1.501	4.549	0.010	89.1100	0.9993

**Table 12:** Slope and intercept parameters with 95% confidence intervals for androgens (GC-MS).

Analytes	Slope (m)	CI_low_(m)	CI_high_(m)	Intercept (q)	CI_low_(q)	CI_high_(q)
DHEA	0.0399	0.0390	0.0408	0.1195	0.1000	0.1389
16α-OHDHEA	0.0317	0.0306	0.0329	0.0435	0.0178	0.0692
D5-ADIOL	0.0792	0.0770	0.0814	0.0916	0.0427	0.1405
D5-ATRIOL	0.0286	0.0265	0.0306	0.1465	0.1008	0.1922
ET	0.0477	0.0466	0.0488	0.0132	−0.0114	0.0377
AN	0.0248	0.0239	0.0257	0.0869	0.0667	0.1070
5α-ADIOL	0.0096	0.0091	0.0101	0.1874	0.1758	0.1990
11β-OHAN	0.0577	0.0526	0.0627	0.1363	0.0237	0.2489

**Table 13:** LOD, LOQ, LLOQ, recovery, and $R^{2}$ for androgens (GC-MS).

Analytes	LOD (μg/mL)	LOQ (μg/mL)	LLOQ (μg/mL)	Recovery (%)	R^2^
DHEA	0.899	2.724	0.010	88.7100	0.9998
16α-OHDHEA	1.491	4.519	0.010	89.6400	0.9993
D5-ADIOL	1.137	3.447	0.010	111.3700	0.9996
D5-ATRIOL	2.947	8.930	0.010	103.6200	0.9974
ET	0.948	2.871	0.010	97.8000	0.9997
AN	1.497	4.536	0.010	101.5400	0.9993
5α-ADIOL	2.233	6.766	0.010	97.0200	0.9985
11β-OHAN	3.599	10.906	0.010	90.5600	0.9961

**Table 14:** Slope and intercept parameters with 95% confidence intervals for estrogens (GC-MS).

Analytes	Slope (m)	CI_low_(m)	CI_high_(m)	Intercept (q)	CI_low_(q)	CI_high_(q)
E3	0.0636	0.0619	0.0654	0.0807	0.0410	0.1204
E2	0.0607	0.0584	0.0630	0.0049	−0.0463	0.0561
E1	0.0625	0.0570	0.0681	0.1231	−0.0012	0.2474

**Table 15:** LOD, LOQ, LLOQ, recovery, and $R^{2}$ for estrogens (GC-MS).

Analytes	LOD (μg/mL)	LOQ (μg/mL)	LLOQ (μg/mL)	Recovery (%)	R^2^
E3	1.149	3.481	0.010	99.1000	0.9996
E2	1.555	4.712	0.010	101.6600	0.9993
E1	3.662	11.097	0.010	114.5100	0.9960

**Table 16:** Repeatability (Rep.) and reproducibility (Repr.) expressed as CV% for urinary progestin metabolites. Analytes: Rep. = Repeatability; Repr. = Reproducibility; Tgt = target ion; Q1 and Q2 = qualifier ions.

Analyte	QC Level	Rep. (%CV)	Repr. (%CV)	Tgt (*m*/*z*)	Q1 (*m*/*z*)	Q2 (*m*/*z*)
17HP	High	11.1	11.8	476	386	364
17HP	Low	8.4	8.8	476	386	364
17HP	Medium	9.7	10.7	476	386	364
P2	High	10.2	11.0	284	346	449
P2	Low	9.9	11.2	284	346	449
P2	Medium	6.0	7.7	284	346	449
PT	High	10.6	10.9	433	343	253
PT	Low	8.5	10.2	433	343	253
PT	Medium	7.5	8.1	433	343	253
PTNE	High	4.7	6.2	449	269	359
PTNE	Low	7.2	7.9	449	269	359
PTNE	Medium	5.6	7.3	449	269	359
5PT	High	5.3	6.7	372	462	267
5PT	Low	9.6	10.9	372	462	267
5PT	Medium	6.8	8.6	372	462	267

**Table 17:** Repeatability (Rep.) and reproducibility (Repr.) expressed as CV% for urinary mineralocorticoid metabolites. Analytes: Rep. = Repeatability; Repr. = Reproducibility; Tgt = target ion; Q1 and Q2 = qualifier ions.

Analyte	QC Level	Rep. (%CV)	Repr. (%CV)	Tgt (*m*/*z*)	Q1 (*m*/*z*)	Q2 (*m*/*z*)
5α-THB	High	6.5	8.4	564	474	384
5α-THB	Low	10.4	10.7	564	474	384
5α-THB	Medium	10.4	11.1	564	474	384
THA	High	8.1	9.0	490	400	431
THA	Low	7.1	8.1	490	400	431
THA	Medium	7.2	8.7	490	400	431
THDOC	High	11.1	12.5	476	188	507
THDOC	Low	11.5	12.8	476	188	507
THDOC	Medium	7.3	8.3	476	188	507
THB	High	5.8	7.5	474	564	384
THB	Low	5.6	7.2	474	564	384
THB	Medium	10.4	11.0	474	564	384

**Table 18:** Repeatability (Rep.) and reproducibility (Repr.) expressed as CV% for urinary glucocorticoid metabolites. Analytes: Rep. = Repeatability; Repr. = Reproducibility; Tgt = target ion; Q1 and Q2 = qualifier ions.

Analyte	QC Level	Rep. (%CV)	Repr. (%CV)	Tgt (*m*/*z*)	Q1 (*m*/*z*)	Q2 (*m*/*z*)
11ketoET	High	10.6	12.3	300	261	405
11ketoET	Low	5.0	6.6	300	261	405
11ketoET	Medium	11.4	12.1	300	261	405
11β-OH-ET	High	7.9	8.3	448	268	358
11β-OH-ET	Low	7.5	9.2	448	268	358
11β-OH-ET	Medium	9.4	10.9	448	268	358
20α DIIDRO-F	High	7.6	8.6	296	243	476
20α DIIDRO-F	Low	6.7	8.5	296	243	476
20α DIIDRO-F	Medium	7.0	8.8	296	243	476
5α-TH-F	High	6.0	7.4	652	562	472
5α-TH-F	Low	5.5	5.8	652	562	472
5α-TH-F	Medium	6.5	6.9	652	562	472
F	High	6.6	7.3	605	515	361
F	Low	11.0	13.0	605	515	361
F	Medium	4.9	5.3	605	515	361
THS	High	9.6	10.5	564	474	384
THS	Low	4.5	4.8	564	474	384
THS	Medium	6.6	8.1	564	474	384
TH-F	High	8.2	10.1	652	472	562
TH-F	Low	9.4	10.8	652	472	562
TH-F	Medium	7.3	8.8	652	472	562
THE	High	9.7	11.6	578	609	488
THE	Low	8.9	10.8	578	609	488
THE	Medium	10.4	11.5	578	609	488
βCORTOL	High	10.6	10.9	343	243	253
βCORTOL	Low	5.6	6.8	343	243	253
βCORTOL	Medium	9.7	11.5	343	243	253
βCORTOLONE	High	9.4	10.3	449	359	269
βCORTOLONE	Low	7.0	7.5	449	359	269
βCORTOLONE	Medium	9.6	11.1	449	359	269
αCORTOL	High	6.3	7.9	343	243	253
αCORTOL	Low	10.4	11.3	343	243	253
αCORTOL	Medium	9.9	11.9	343	243	253
αCORTOLONE	High	6.6	7.2	449	359	269
αCORTOLONE	Low	11.4	12.9	449	359	269
αCORTOLONE	Medium	9.3	11.1	449	359	269

**Table 19:** Repeatability (Rep.) and reproducibility (Repr.) expressed as CV% for urinary estrogen metabolites. Analytes: Rep. = Repeatability; Repr. = Reproducibility; Tgt = target ion; Q1 and Q2 = qualifier ions.

Analyte	QC Level	Rep. (%CV)	Repr. (%CV)	Tgt (*m*/*z*)	Q1 (*m*/*z*)	Q2 (*m*/*z*)
17β-ESTRADIOLO	High	5.7	7.4	531	441	459
17β-ESTRADIOLO	Low	6.4	8.3	531	441	459
17β-ESTRADIOLO	Medium	10.9	12.6	531	441	459
E3	High	5.7	6.6	504	386	311
E3	Low	4.6	5.5	504	386	311
E3	Medium	9.3	9.9	504	386	311
E1	High	8.1	8.8	371	340	356
E1	Low	6.4	7.7	371	340	356
E1	Medium	11.3	11.8	371	340	356

**Table 20:** Repeatability (Rep.) and reproducibility (Repr.) expressed as CV% for urinary androgen metabolites. Analytes: Rep. = Repeatability; Repr. = Reproducibility; Tgt = target ion; Q1 and Q2 = qualifier ions.

Analyte	QC Level	Rep. (%CV)	Repr. (%CV)	Tgt (*m*/*z*)	Q1 (*m*/*z*)	Q2 (*m*/*z*)
11β-OH-AN	High	8.3	9.9	360	270	213
11β-OH-AN	Low	8.4	9.5	360	270	213
11β-OH-AN	Medium	6.6	7.8	360	270	213
16α-OH-DHEA	High	8.8	10.0	446	266	356
16α-OH-DHEA	Low	5.4	6.8	446	266	356
16α-OH-DHEA	Medium	10.0	11.2	446	266	356
5α-ANDROSTANEDIOL	High	8.1	9.2	421	379	241
5α-ANDROSTANEDIOL	Low	5.9	6.9	421	379	241
5α-ANDROSTANEDIOL	Medium	9.9	11.7	421	379	241
D5-ATRIOL	High	9.6	11.5	432	522	417
D5-ATRIOL	Low	7.1	8.9	432	522	417
D5-ATRIOL	Medium	8.6	9.4	432	522	417
AN	High	7.2	8.7	360	270	213
AN	Low	6.9	8.3	360	270	213
AN	Medium	5.9	7.3	360	270	213
D5-ADIOL	High	7.1	7.7	344	434	239
D5-ADIOL	Low	10.6	11.2	344	434	239
D5-ADIOL	Medium	10.9	12.5	344	434	239
DHEA	High	10.7	11.3	358	389	268
DHEA	Low	5.2	5.9	358	389	268
DHEA	Medium	11.0	12.3	358	389	268
ET	High	10.3	11.2	360	270	213
ET	Low	6.5	7.9	360	270	213
ET	Medium	10.9	11.5	360	270	213

## Data Availability

The original contributions presented in the study are included in the article, further inquiries can be directed to the corresponding author.

## Analytes

The following Analytes are used in this manuscript:

GC-MS	Gas Chromatography–Mass Spectrometry
LOD	Limit of Detection
LLOQ	Lower Limit of Quantification
QC	Quality Control
CV%	Coefficient of Variation (Percent)
CAH	Congenital Adrenal Hyperplasia
IS	Internal Standard
SPE	Solid Phase Extraction
SUD	Sigmatrix Urine Diluent
MOX	Methoxyamine
ICH	International Council for Harmonisation
Rep.	Repeatability
Repr.	Reproducibility
TIC	Total Ion Chromatogram
RT	Retention Time
Pregnanediol	P2
Pregnentriol	5PT
17OH-Pregnanolone	17HP
Pregnantriol	PT
Pregnantriolone	PTONE
TH-11Desoxicorticosterone	THDOC
TH-11Dehydrocorticosterone	THA
5α-Tetrahydrocorticosterone	5αTHB
Tetrahydrocorticosterone	THB
TH-11Deoxycortisol	THS
Cortisol	F
Tetrahydrocortisol	THF
5α-Tetrahydrocortisol	5α-THF
β-Cortol	β-Cortolo
α-Cortol	α-Cortolo
20α-Dihydrocortisol	20α-DHF
11β-Hydroxyetiocholanolone	11β-OHET
Tetrahydrocortisone	THE
α-Cortolone	α-Cortolone
β-Cortolone	β-Cortolone
11-Ketoetiocholanolone	11ketoET
Dehydroepiandrosterone	DHEA
16α-Hydroxy-DHEA	16α-OHDHEA
5-Androstene-3β,17β-diol	D5-ADIOL
Androstenetriol	D5-ATRIOL
Etiocholanolone	ET
Androsterone	AN
5α-Androstanediol	5α-ADIOLO
11β-Hydroxyandrosterone	11β-OHAN
Estriol	E3
17β-Estradiol	E2
Estrone	E1
